# Integrated In Silico and Experimental Validation of Antrocin as a Plant-Derived Multi-Target Therapeutic for BRAF/MEK/PI3K-Driven Colorectal Cancer

**DOI:** 10.3390/ijms26188780

**Published:** 2025-09-09

**Authors:** Jian-Syun Chen, Chioma Grace Enwolo-Chibueze, Harold Arnold Chinyama, Cheng-Ta Lai, Ifeyinwa Chioma Ezeala, Po-Yang Huang, Alexander T. H. Wu, Yan-Jiun Huang

**Affiliations:** 1 Division of Colon and Rectal Surgery, Department of Surgery, MacKay Memorial Hospital, Taipei 104217, Taiwan; manchette.4320@mmh.org.tw (J.-S.C.); iamctlai.4987@mmh.org.tw (C.-T.L.); 2 Department of Medicine, MacKay Medical University, New Taipei City 252005, Taiwan; 3 Ph.D. Program for Cancer Biology and Drug Discovery, College of Medical Science and Technology, Taipei Medical University, Taipei 11031, Taiwan; m654113004@tmu.edu.tw; 4 Graduate Institute of Biomedical Informatics, College of Medical Science and Technology, Taipei Medical University, Taipei 11031, Taiwan; d610113001@tmu.edu.tw; 5 Department of Pharmaceutical and Medicinal Chemistry, Faculty of Pharmaceutical Sciences, University of Nigeria Nsukka, Nsukka 410001, Nigeria; ifeyinwa.adaka@unn.edu.ng; 6 The Ph.D. Program of Translational Medicine, College of Medical Science and Technology, Taipei Medical University, Taipei 11031, Taiwan; boyang0321@gmail.com (P.-Y.H.); chaw1211@tmu.edu.tw (A.T.H.W.); 7 Clinical Research Center, Taipei Medical University Hospital, Taipei Medical University, Taipei 11031, Taiwan; 8 Graduate Institute of Medical Sciences, National Defense Medical Center, Taipei 11490, Taiwan; 9 Taipei Heart Institute, Taipei Medical University, Taipei 11031, Taiwan; 10 Department of Surgery, School of Medicine, College of Medicine, Taipei Medical University, Taipei 11031, Taiwan; 11 Division of Colorectal Surgery, Department of Surgery, Taipei Medical University Hospital, Taipei Medical University, Taipei 11031, Taiwan; 12 Division of General Surgery, Department of Surgery, Taipei Medical University Hospital, Taipei Medical University, Taipei 11031, Taiwan

**Keywords:** colorectal cancer (CRC), BRAF/MEK/PI3K oncogenic signature, Antrocin, drug resistance, bioinformatics, multi-target therapy

## Abstract

Colorectal cancer (CRC) remains a leading cause of cancer-related death worldwide, with resistance to targeted therapies presenting a significant clinical challenge. This study combines computational and experimental methods to identify and validate Antrocin, a natural sesquiterpene lactone, as a potential multi-target inhibitor of the BRAF/MEK/PI3K oncogenic pathway in CRC. Differential gene expression and mutational analyses were performed using public datasets (TCGA, TNMplot, GEPIA2, GSCA, PANDA, and cBioPortal) to assess the prevalence and clinical significance of BRAF, MEK, and PI3K alterations in CRC. In silico molecular docking, using AutoDock Vina, predicted strong binding affinities of Antrocin to BRAF (ΔG = −8.5 kcal/mol), MEK (ΔG = −7.3 kcal/mol), and PI3K (ΔG = −6.9 kcal/mol), comparable to those of FDA-approved inhibitors for BRAF (Dabrafenib), MEK (Trametinib), and PI3K (Alpelisib). Drug-likeness and ADME properties were evaluated via SwissADME and ADMETlab, supporting Antrocin’s potential as a drug candidate. In vitro assays using HCT116 and RKO CRC cell lines validated that Antrocin treatment suppressed cell viability, spheroid formation, and migration, accompanied by reduced expression levels of the oncogenic BRAF/MEK/PI3K signaling pathway. Antrocin-treated tumor-conditioned medium experiments demonstrated Antrocin’s ability to reduce the differentiation of cancer-associated fibroblasts and the polarization of M2 macrophages. Preclinical mouse xenograft experiments demonstrated a delay in tumor growth following treatment with Antrocin. These results suggest that Antrocin, identified through computational screening and validated experimentally, could be a promising multi-target agent to overcome therapy resistance in CRC.

## 1. Introduction

Colorectal cancer (CRC) accounts for 9.6% of all cancers and is ranked as the third most prevalent cancer and the second most common cause of cancer deaths in the world. A gradual increase from 63% to 73% of the incidences and mortality rates, respectively, is expected by the year 2040, presenting colorectal cancer as a global health burden [1]. Due to late symptomatic manifestation, CRC is often diagnosed in advanced stages, complicating the curative efforts and consequently shortening the overall survival. Current treatment options for colorectal cancer comprise surgery for localized tumors, radiation therapy, chemotherapy, targeted therapy, and immunotherapy [2,3]. Resistance to treatment in CRC is prevalent and is closely associated with tumor heterogeneity and genetic mutations [2,4]. These have resulted in 64% patient overall survival, down to 14% in metastatic types, 29% of CRC recurrence within five years of treatment, and high financial strain, outcomes from the late detection rate and aforementioned resistance trails [5,6,7]. This escalating incidence and mortality, coupled with inherent resistance mechanisms, underscore the growing urgency for novel therapeutic interventions.

CRC therapy faces challenges due to multiple dysregulations of oncogenic signaling pathways. Our data mining and bioinformatics analyses identified an oncogenic signature involving the BRAF, MEK, and PI3K pathways. Dysregulation of this signature promotes CRC progression and resistance [8,9,10]. Genomic profiling shows BRAF (BRAF-V600E mutant), MEK (MAP2K1), and PI3K (PIK3CA) mutations are common in CRC and associated with aggressive tumors, metastasis, and poor prognosis [11,12,13,14]. Targeted inhibitors like Dabrafenib, Trametinib, and Alpelisib show limited success in CRC due to resistance from activation of parallel pathways and feedback loops. Current single-target therapies rarely produce lasting responses, emphasizing the need for multi-targeted strategies [12,13,14,15,16,17,18].

Natural sources such as *Antrodia cinnamomea* (AC) offer promising multi-targeted agents. Used by Taiwanese indigenous people for centuries, AC exhibits anti-inflammatory, anti-diabetic, anti-cancer, antioxidant, and immunomodulatory effects. Notably, a bioactive component of AC, Antrocin, has been shown to suppress tumorigenesis in the prostate, bladder, breast, and lung by inhibiting JAK2/STAT3, PI3K/Akt/MAPK, and β-catenin mTOR/GSK-3β/NF-κB signaling pathways [19,20]. These reported anticancer studies of Antrocin provide a strong rationale for its potential as a multi-target inhibitor for CRC.

The present study employed a comprehensive integrated approach. First, an in silico exploration was conducted to identify and characterize the BRAF/MEK/PI3K oncogenic signature in CRC, demonstrating how mutations and differential expression remodel the tumor microenvironment and impact overall patient survival. This computational analysis positioned Antrocin as a promising multi-targeted therapeutic candidate in CRC. Second, experimentally, this study validated Antrocin’s ability to simultaneously inhibit BRAF, MEK, and PI3K genes present in HCT116 and RKO cell lines, hence its broader anti-tumor effects in CRC, and compared its putative efficacy with that of FDA-approved standard inhibitors. This synergistic approach, combining in silico methods for rapid hypothesis generation with experimental validations, was designed to increase confidence in Antrocin’s therapeutic potential and advance its development as a novel agent for the management of colorectal cancer by targeting the BRAF/MEK/PI3K tumor proliferative axis and overcoming drug resistance.

## 2. Results

### 2.1. BRAF/MEK/PI3K Is Overexpressed in the Pan-Cancer and Promotes Tumor Proliferation and Metastasis in CRC

**Figure 1:**
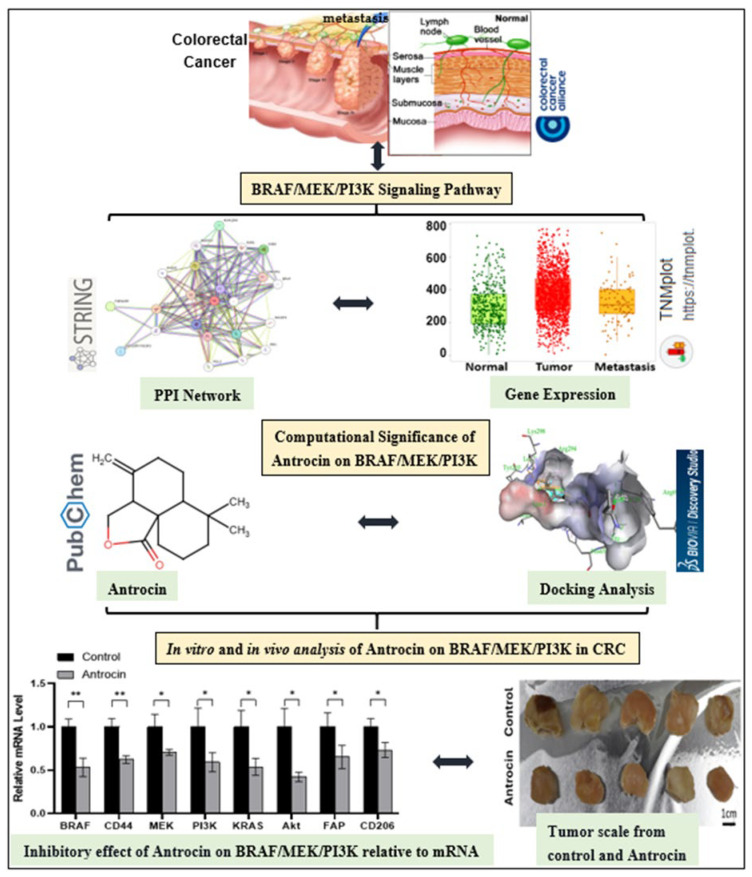
Workflow illustrating sequence from bioinformatics analysis of BRAF/MEK/PI3K to biological investigations of Antrocin inhibitory effects on CRC signaling pathway.Where asterisk (*) indicate level significance in Antrocin treated group compared to control group (* *p* < 0.05 and ** *p* < 0.01).

**Figure 2:**
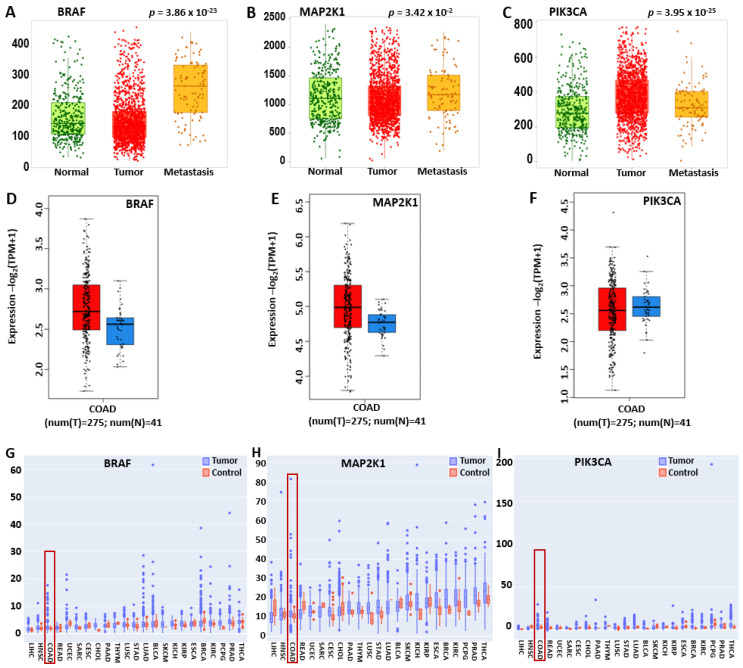
Overexpression of the BRAF/MEK/PI3K oncogenic signature in pan-cancer, tumor, and metastatic CRC. (**A**–**C**) Box plots showing high BRAF, MEK, and PI3K expression in tumor and metastatic tissues compared to normal tissues. (**D**–**F**) GEPIA2 DEG results show overexpression of BRAF/MEK/PI3K in tumor tissues (red) compared to normal tissues (blue) with *p* < 0.05. (**G**–**I**) Depicts BRAF/MEK/PI3K overexpression in pan-cancer via PANDA transcriptomic analysis.

Validation of BRAF/MEK/PI3K expression in CRC (Figure 1) on TNMplot using gene chip data revealed the statistically significant overexpression of BRAF in metastatic tissues compared to normal tissues (Figure 2A). However, its high expression in tumor samples was slightly lower than in metastatic samples, indicating that BRAF’s overexpression is associated with tumorigenesis, tumor aggressiveness, and progression to advanced stages. Tumor and metastatic tissues exhibited higher PIK3CA expression compared to normal tissues (Figure 2C). Tumor samples exhibited slightly higher expression than metastatic samples, suggesting that PI3K3CA is involved in tumorigenesis (As shown, MAP2K1 and PIK3CA expressions did not significantly increase in metastasis (Figure 2B,C).

Further validation using GEPIA2 showed high expression of BRAF, MEK, and PI3K in CRC at *p* < 0.05 (Figure 2D–F). In addition, we analyzed the expression of BRAF, MEK, and PI3K in pan-cancer on PANDA, with a particular interest in their expression levels in CRC. The pan-cancer expression levels were analyzed from PANDA transcriptome data featuring patient status in colorectal cancer samples highlighted in red box, where BRAF was overexpressed compared to adjacent normal tissues, with a *p*-value significance code of <0.05 (Figure 2G). MAP2K1 had a similar expression at *p*-value < 0.001, and PIK3CA had the least *p*-value of 0.127, which may be due to the fact that the data were analyzed based on patient status at the collection time (Figure 2H,I). This result is presented in Figure 2.

### 2.2. BRAF/MEK/PI3K Cancer Hallmark and CRC Correlation Cell Type Activities

**Figure 3:**
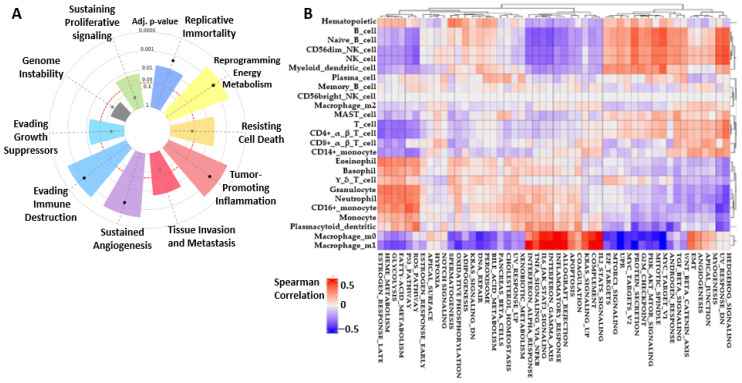
Interplay correlation activities between colorectal cancer hallmarks and immune components that are key players in the tumor microenvironment (TME). In the heatmap, red color indicates intense correlation, while blue shows less correlation (**A**) BRAF/MEK/PI3K tumorigenic cancer hallmark event intensities are measured according to their *p*-values and distribution extents in dots where expressions above the red circle margin indicate significant *p*-values < 0.05. (**B**) CRC hallmark pathways in correlation with immune signals involved in TME.

The aggressiveness of any cancer-related gene is embedded in its hallmark of activities; hence, below we used the CancerHallmark platform (https://cancerhallmarks.com/) accessed on 22 June 2025, where 6763 samples were analyzed in the integrated cancer hallmark gene set (Figure 3A). It is observed that BRAF/MEK/PI3K signaling encompassed all cancer hallmarks, which gave insights on how this axis can be aggressive in tumor growth and metastasis. Signals like sustaining proliferative signaling, evading growth suppressors, immune destruction, sustaining angiogenesis, tumor-promoting inflammation, resisting cell death, replicative immortality, reprogramming energy metabolism, tissue invasion, and metastasis were significant within *p*-values of 0.001 < *p* < 0.05 in Table 1. Only MAP2K1 expressed genomic instability, though at a very low *p*-value. Studies have shown that using combination therapies targeting [21,22,23] BRAF/MEK/PI3K will go a long way to overcome tumor resistance mechanisms. Other hallmarks were analyzed in detail and in correlation with immune infiltration in colorectal cancer to ascertain which biological pathway is most upregulated or downregulated in COAD using Spearman correlation (Figure 3B) [24] as a measure of association (https://panda.bio.uniroma2.it accessed on 23 June 2025). Red indicates positive correlation, while blue shows little correlation, being within the −0.5–0.5 range. One can relate from this that most of the hallmarks in relation to immune highlights are in positive correlation with colorectal cancer, which has made the disease a difficult one to manage with 75% intermediate and 45% high risk. Major immune components like NK cells, macrophages, neutrophils, and dendritic cells, which proliferate CRC development, were in positive correlation with inflammatory response, angiogenesis, the PIK_AKT_MTOR axis, KRAS, and IL6_JAK_STAT3 signaling, amongst others. RTK-RAS, TGF-β, and PI3K (Figure 3B), which are also commonly expressed hallmarks in CRC, are explained in the literature [25,26].

### 2.3. BRAF/MAP2K1/PIK3CA Gene Ontology (GO) and KEGG Pathway

**Figure 4:**
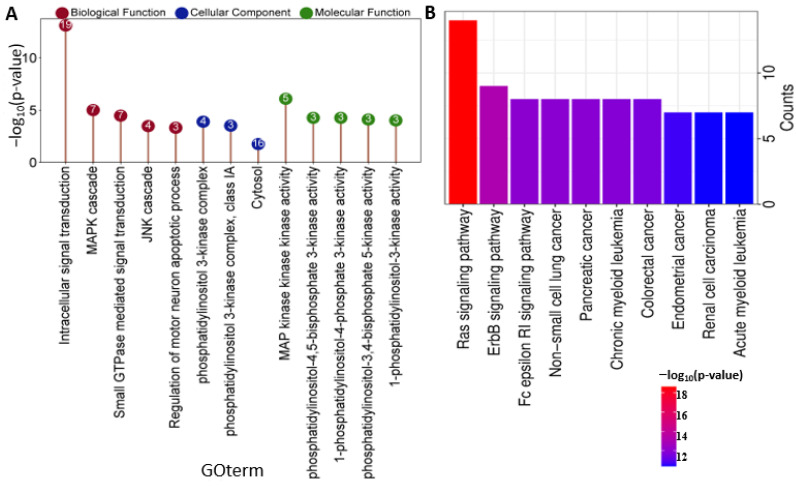
Gene Ontology of the BRAF/MEK/PI3K axis encompassing its protein–protein network and signaling pathways showcasing the biological function (BF) and molecular functions (MFs) at a false discovery rate (FDR) of less than or equal to 0.001 and cellular component (CC) at <0.05, which explains the rationale behind the proliferative potentials of BRAF/MEK/PI3K in CRC (**A**) Lollipop plot of BRAF/MEK/PI3K in BF/CC/MF GO process where the numbers highlighted indicates the number of genes involved in each GO term (**B**) Bar chart showing the KEGG pathway of the BRAF/MEK/PI3K axis.

The Gene Ontology (GO) explains the roles of genes and their products in a biological context across multiple species. The GO functions of BRAF/MEK/PI3K encompass a lot of activities shown in the literature highlighted in CRC development [27]. These activities involve transcellular signal transduction (most pronounced –log10(p) value and 19 gene counts), MAPK cascade, JNK cascade, MAP kinase activity, phosphatidylinositol activities, and related cellular components (Figure 4A). The KEGG platform illustrates different pathways that depict the aggressiveness of BRAF/MEK/PI3K signaling and various cancer types implicated (Figure 4B). These include Ras signaling, ErbB signaling, NSCLC, AML, pancreatic cancer, colorectal cancer, and renal cancer as a result of treatment resistance mechanisms from the BRAF/MEK/PI3K axis [10]. This is a confirmation that BRAF/MEK/PI3K oncogenic signaling involves intrinsic activities highly involved in colorectal cancer progression. The information was obtained from https://string-db.org/ platform version 12.0, while the illustration was performed using https://www.bioinformatics.com.cn/en software PLoS ONE 18 version (all accessed on 23 June 2025).

### 2.4. Mutations in BRAF/MEK/PI3K and Their Expression Changes Promote CRC Tumor Growth

**Figure 5:**
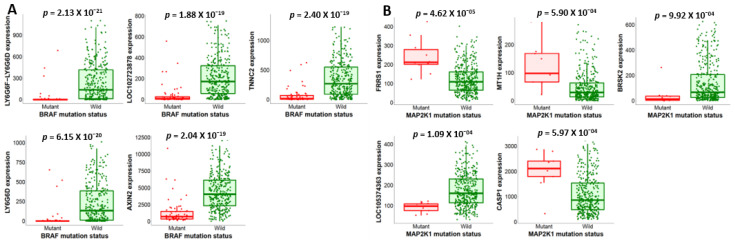

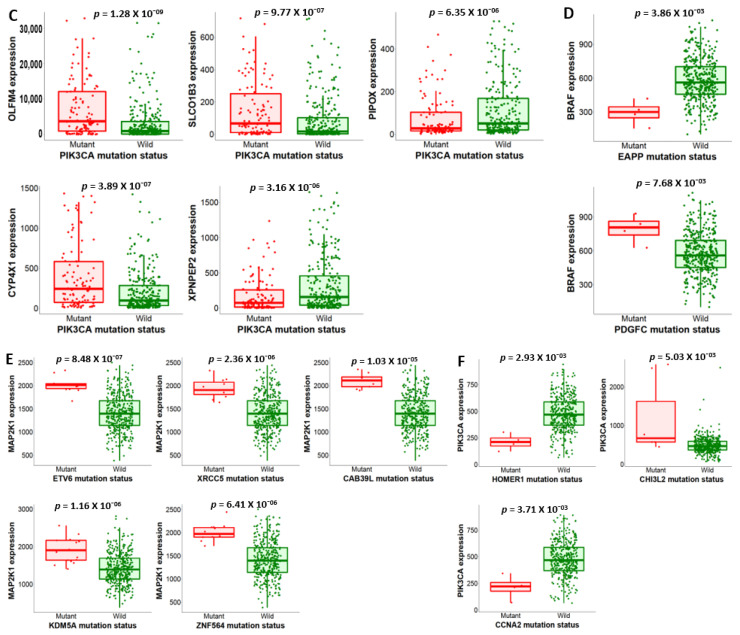
Association between BRAF/MEK/PI3K mutations and colon adenocarcinoma growth, survival, and metastasis. (**A**–**C**) highlight the top 5 genes affected by these mutations at *p* < 0.01, FDR 5%, and 2.0-fold change. BRAF mutation lowered TNNC2, AXIN2, LY6G6D, LOC102723878, and LY6G6F-LY6G6D but increased FRRS1, MT1H, and CASP1 with MEK mutation and overexpressed OLFM4, CYP4X1, and SLCO1B3 with PI3K mutation, while XPNPEP2 and PIPOX were reduced. (**D**–**F**) Differential expression with the top mutated genes at *p* < 0.01. BRAF overexpression is linked to PDGFC mutation but lowered by EAPP; MEK upregulation is linked to ETV6, KDM5A, XRCC5, ZNF564, and CAB39L mutations. PI3K downregulation correlated with CCNA2 and HOMER1 mutations, and high PI3K expression correlated with CHI3L2 mutations.

The top five genes whose expression is affected by the mutation status of BRAF, MEK, and PI3K were analyzed at the genotypic level on the muTarget platform with *p* < 0.01, a 2.0-fold change cut-off, and an FDR of 5% (Figure 5A–E). BRAF mutations downregulated AXIN2 (Figure 5A), an important tumor suppressor and regulator of the Wnt/β-catenin signaling pathway in CRC [28,29,30]. AXIN2 downregulation leads to a dysregulated Wnt/β-catenin pathway whose over activation leads to aberrant cell proliferation and tumor growth in CRC. FRRS1 regulates iron homeostasis and the subsequent cellular processes that require iron, such as DNA synthesis in proliferating cells [31]. Its upregulation is closely associated with MEK mutation in CRC (Figure 5B). This upregulation promotes rapid cell proliferation and tumor growth. BRSK2 downregulation by the MEK mutant gene (Figure 5B) results in dysregulated cell proliferation and tumor growth, as BRSK2 regulates the G1/S transition [32,33]. In addition, CASP1 and MT1H genes were upregulated in CRC-harboring MEK mutants (Figure 5B). PI3K mutation upregulates OLFM4, which promotes tumor cell adhesion and proliferation and suppresses apoptosis in CRC [34,35]. SLCO1B3 and CYP4X1 play an important role in drug and substance metabolism and are all upregulated by PI3K mutants (Figure 5C,D) [36,37,38]. We analyzed the relationship between BRAF, MEK, and PI3K expression changes and the mutation status of the top 5 genes at the target level using the muTarget tool. BRAF upregulation correlates with PDGFC mutation, which promotes tumor growth, survival, angiogenesis, and metastasis (Figure 5D). BRAF downregulation links to EAPP mutants affecting tumor growth and patient outcomes. High MEK expression is associated with ETV6, KDM5A, XRCC5, ZNF564, and CAB39L mutants. PI3K upregulation relates to mutant CHI3L2, while its downregulation links to CCNA2 and HOMER1 mutants (Figure 5E,F).

### 2.5. BRAF/MEK/PI3K Oncogenic Signature Promotes Tumor Aggressiveness, Therapy Resistance, and Poor Overall Survival

**Figure 6:**
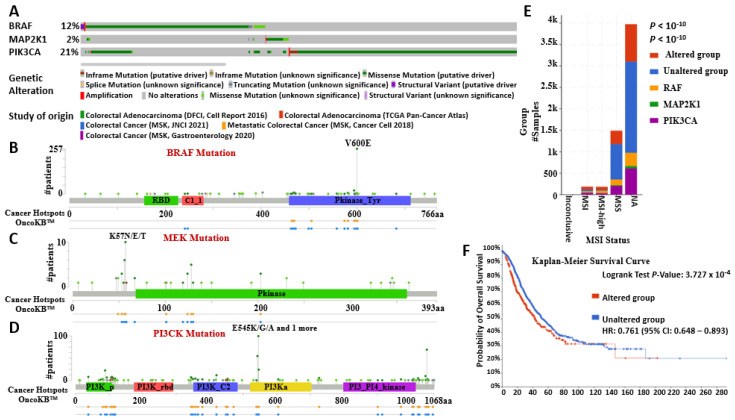
BRAF/MEK/PI3K mutations promote tumor aggressiveness, therapy resistance, and poor overall survival in CRC. (**A**) Bar chart comprising the mutation types and their frequency in BRAF (12%), MEK (2%), and PI3K (21%) in the five CRC studies. (**B**–**D**) Lollipop plots of the color-coded driver mutations across the BRAF, MEK, and PI3K protein domains in CRC patients. (**E**) Stacked-bar chart of the microsatellite mutations comprising MSI, MSI-H, and MSS in altered (red) and unaltered (blue) groups, BRAF (orange), MEK (green), and PI3K (purple). (**F**) Kaplan–Meier survival curve showing the low overall survival of CRC patients who have a mutated BRAF/MEK/PI3K oncogenic signature (red).

This analysis revealed 481 driver mutations and 74 VUS on BRAF (12%), 54 driver mutations on MEK (2%), and 921 driver mutations on PI3K (21%). Missense mutations were most common, followed by truncating, in-frame, and splice mutations. We found multiple hotspots and driver mutations in the BRAF/MEK/PI3K pathway (Figure 6A), which are crucial for tumor progression, growth, survival, and therapy resistance in CRC and other tumors like melanoma and breast cancer, due to abnormal kinase activation and pathways. [39,40]. In addition to the V600E (BRAF), K57N/E/T (MEK), and E545K/G/A (PI3K) mutations (Figure 6B–D), our analysis revealed other driver mutations such as N581, L505, G469A/V, and K601ER38H/C in the BRAF gene; P471, P124, E203K, F53L/V, and E102_I103del in the MEK gene; and H1047R, R357L/Q, G118D, and E726 in the PI3K gene driving growth signaling and survival pathways in CRC, melanoma, breast cancer, and other solid tumors [39,40]. Mutated BRAF/MEK/PI3K contributed to the high-level microsatellite instability (MSI-H), MSI, and microsatellite stable (MSS) phenotypes [41,42]. However, there was an increased number of mutated BRAF/MEK/PI3K samples (352) within the MSS CRC, which has a poor prognosis compared to the MSI-H (93 samples) (Figure 6E) [41,42,43]. Furthermore, there is a significant reduction in the overall survival in CRC patients with a mutated BRAF/MEK/PI3K oncogenic signature (Figure 6F), underlining its role in tumor aggressiveness and resistance to therapy. These results were analyzed on cBioPortal for Cancer Genomics.

### 2.6. BRAF/MEK/PI3K Correlation and Functional Enrichment in Colorectal Cancer

**Figure 7:**
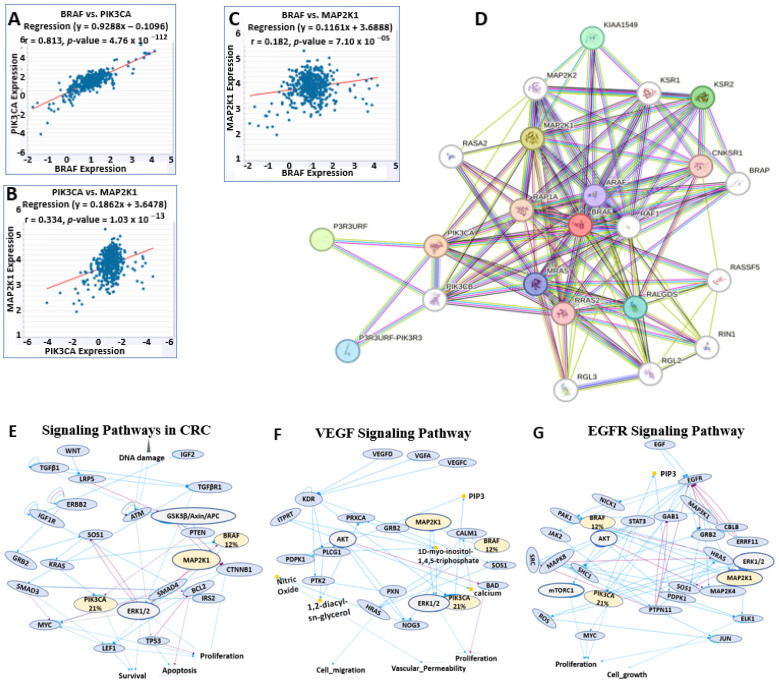
The BRAF/MEK/PI3K oncogenic signature is highly correlated and integral in proliferation, growth, and survival signaling pathways. (**A**–**C**) shows scatter plots comprising the correlation of BRAF/MEK/PI3K in COAD with the correlation coefficient (r) of 0.813, 0.334, and 0.182 analyzed from the 471 samples of the ENCORI project, with *p* < 0.05. (**D**) Interaction and enrichment network of the BRAF/MEK/PI3K in CRC. It comprises the signaling pathways in colorectal cancer. (**E**–**G**) signaling pathways in CRC, the VEGF signaling pathway, and the EGFR signaling pathway comprise BRAF/MEK/PI3K with genomic alterations, where blue line arrow indicates upregulation, red line arrow shows down regulation and yellow dot depict quantity.

The analysis on the starBase software 3, showed a highly correlated BRAF/MEK/PI3K oncogenic signature in CRC among 471 samples analyzed, with the BRAF/PI3K interaction exhibiting a strong correlation with a correlation coefficient (r) of 0.813 (Figure 7A). At the same time, MEK/PI3K had an r of 0.334 (Figure 7B), and BRAF/MEK had an r of 0.182 (Figure 7C), with all graphs achieving significant *p*-values. The RNA-seq expression values for these interactions were transformed and normalized by log2 (Fragments Per Kilo base of Transcript per Million mapped reads (FPKM) + 0.01). We observed a similar correlation pattern in the network and enrichment analysis on the STRING software 12.0, where BRAF, MEK, and PI3K showed varying degrees of interactions with other genes, such as KSR1 and KSR2, which activate Raf/MEK/ERK pro-tumorigenic signaling in RAS-dependent cancers [44,45]; RALGDS, a mediator of KRAS oncogenic signaling [46]; and MAP2K2, which instigates the MAPK/ERK pathway upregulated in tumors more than normal tissue in colorectal cancer studies [47,48] (Figure 7D). Furthermore, notable genetic alterations to the BRAF/MEK/PI3K signature resulted in a dysregulated VEGF signaling pathway, EGFR signaling pathway involving BRAF 12% and PIK3CA 21% alterations together with other genes causing cell proliferation, migration, growth, and survival signaling pathways, such as the RAS pathway, in colorectal cancer (Figure 7E–G). In this signaling, KRAS upregulates BRAF and PIK3CA activities (Figure 7E), MAP2K1 upregulates BRAF and ERK1/2 effects, PIK3CA increase quantity of PIP3 (Figure 7F) while EGFR increases the activities of BRAF, PIK3CA and MAP3K1 which in turn upregulates MAP2K1 effect (Figure 7G). These pathways were analyzed in cBioPortal for Cancer Genomics version 6.3.6, within a *p*-value of 1.16 × 10^−6^.

### 2.7. BRAF/MEK/PI3K Overexpression Promotes the Recruitment of Pro-Tumor Immune Cells in the CRC Tumor Microenvironment

**Figure 8:**
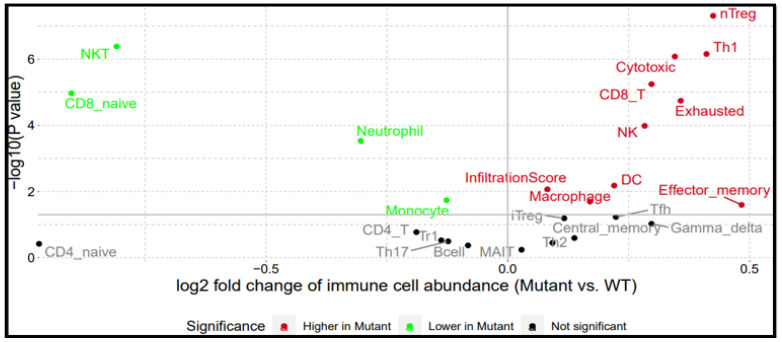
BRAF/MEK/PI3K overexpression promotes the recruitment of pro-tumor immune cells in colorectal cancer. A hypothesis that this proliferative protein axis is shown to significantly increase macrophages, NK cells, CD8_T, and dendritic cells (DCs) (which are immune components responsible for CRC tumor microenvironment activities) more in mutant than wild types.

Gene Set Cancer Analysis revealed that the overexpression of the BRAF/MEK/PI3K oncogenic signature in CRC directly correlates with the recruitment of pro-tumor immune cells into the TME (https://guolab.wchscu.cn/GSCA/#/) accessed on 24 June 2025 (Figure 8). This analysis was based on single nucleotide variants (SNVs) and immune cell abundance, comparing mutants and wild type (WT). Although mutant models had lower neutrophil, monocyte, NKT, and CD8_naive levels, there was a stronger positive correlation between TME and BRAF/MEK/PI3K signaling, with high expression of macrophages, NK cells, DCs, memory cells, infiltration score, and Tregs in CRC mutants compared to wild-type. These are involved in increased tumor proliferation, growth, aggressiveness, immune suppression, tumorigenesis, angiogenesis, and metastasis [49,50,51,52,53,54,55]. Consequently, the tumor microenvironment has a supportive stroma modulated by the recruited pro-tumor immune cells that allows the tumor to evade the anti-tumor immune response, resulting in poor prognosis and overall survival [49,50,51,52,53,54,55]. This result is in tandem with Figure 2B, where high immune signals were seen in correlation with the CRC hallmark.

### 2.8. Analysis of the Presence of BRAF/MEK/PI3K in a Cancerous Colon and Its Expression in CRC Cell Lines

**Figure 9:**
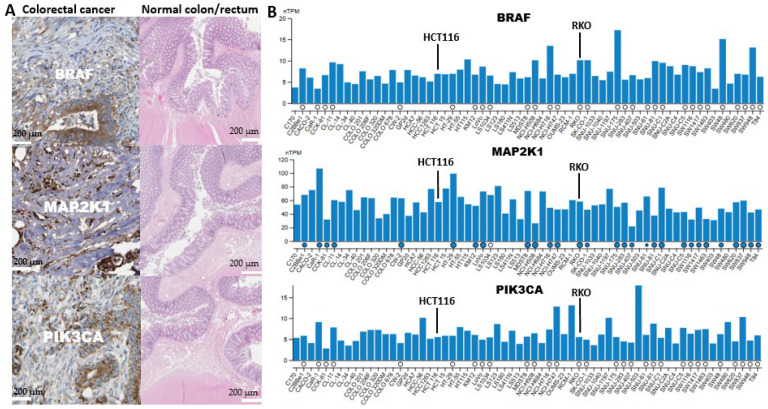
Highlighting the tissue expression effects of BRAF/MEK/PI3K in CRC compared to normal cells, this illustration explains the potentials of this axis to develop cancerous lesions in the colon as obtained from patient immunohistochemistry data published in the human protein atlas data page. (**A**) BRAF/MEK/PI3K proliferative illustrations in cancerous colon vs. normal colon at 200 μm scale bar (**B**) An expression depicting that BRAF/MEK/PI3K are highly expressed in different CRC cell lines while highlighting HCT116 and RKO cells utilized in this study where blue dots indicates gene mutation and white dot no mutation.

This study used the human protein atlas website (https://www.proteinatlas.org/ (accessed on 28 June 2025)) to obtain the information on BRAF/MEK/PI3K tissue and cell line expression, with patient codes: BRAF (CAB004552), MAP2K1 (CAB080093), and PIK3CA (HPA009985) all indicating high staining intensity >76% (Figure 9A). From the histopathological illustrations, it is evident that alterations to the BRAF, MEK, and PI3K genes disrupt the normal morphology of the colon and rectum, resulting in cancerous growth compared to the normal counterparts (Figure 9A), where antibody staining indicated more intense coloration in cancerous cells than normal cells. Several studies have illustrated the importance of BRAF/MEK/PI3K inhibition to combat CRC due to its high tumorigenic potential [12,21]. Given this, we examined the expression of BRAF/MEK/PI3K in CRC-related cell lines to test this hypothesis (Figure 9B). It was observed that these genes were elevated in most cell lines, indicating their high involvement in CRC. Also, there was high expression of these genes in the most aggressive cell lines in colorectal cancer, which include HCT116, HT29, RKO, SW620, SW1417, CL40, and CW2, and are characterized by high malignancy, enhanced invasiveness, advanced stage model, and metastasis [56,57,58].

### 2.9. High BRAF/MEK/PI3K Expression Is Associated with Multi-Drug Resistance in CRC

**Figure 10:**
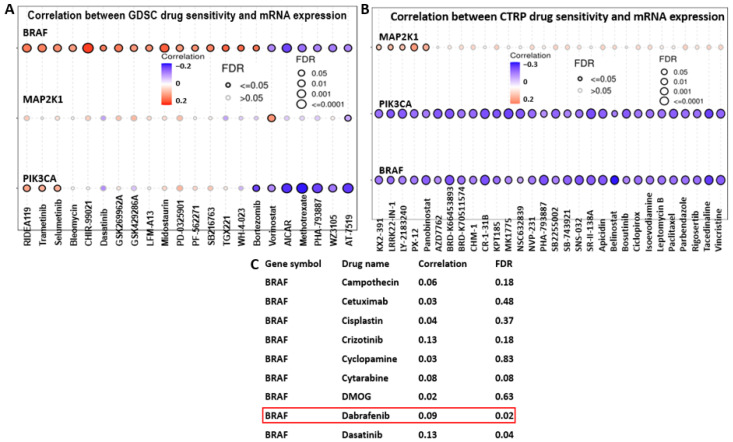
BRAF/MEK/PI3K overexpression is associated with multidrug resistance in colorectal cancer. (**A**,**B**) are bubble plots comprising drug sensitivity profiles of BRAF/MEK/PI3K in CRC, showing multi-drug resistance of these genes in orange bubbles. The blue bubbles depict the sensitivity of the genes to the drugs. (**C**) is an excerpt of BRAF sensitivity to drugs, comprising its low sensitivity to Dabrafenib highlighted in red box, analyzed on the GDSC database.

Analysis of BRAF, MEK, and PI3K expression and drug sensitivity in CRC was achieved using GSCA, which used CTRP and GDSC databases. Results are shown in bubble plots for the top thirty FDA-approved drugs, with correlations in orange and blue (Figure 10A,B). In addition, upregulation of BRAF, PI3K, and MEK is associated with resistance to selective MEK inhibitors, such as Refametinib (RDEA119), Mirdametinib (PD-0325901), and Selumetinib [59,60] (Figure 10A). Deep colors indicate strong correlation; bubble size reflects FDR significance, with black borders for FDR ≤ 0.05. A snippet of the drug sensitivity profile of BRAF from the GDSC profile of BRAF sensitivity. Overexpression of these genes is linked to drug resistance and poor prognosis. BRAF overexpression promotes resistance to Trametinib and Dabrafenib (Figure 10A–C) targeting V600E or V600K mutations in BRAF [61]. MEK also showed resistance to Vorinostat and Panobinostat, which are histone deacetylase inhibitors (HDAC) [62,63,64].

### 2.10. Antrocin Meets All Drug-Likeness, Absorption, Distribution, Metabolism, and Excretion (ADME) Properties of Small-Molecule Drugs

**Figure 11:**
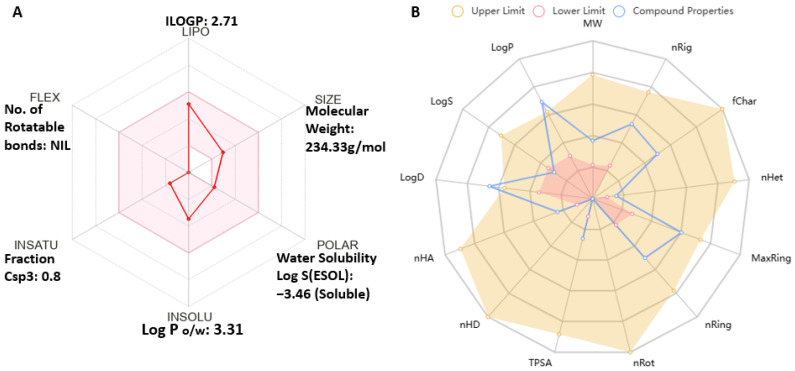
Antrocin met the drug-likeness, pharmacokinetics (PKs), ADME, and toxicity evaluation standards for small compounds. (**A**) A bioavailability radar illustrates the physicochemical properties of Antrocin evaluated using SwissADME version 5.0 where the red connecting dots shows Antrocin druglikeness seated within the acceptable web. (**B**) Pharmacokinetics detailing the profile of Antrocin from ADMET-lab software 3.0 where red dot spider web indicates lowest acceptable limit, orange dot radar shows upper limit ADMET and blue radar signifies Antrocin ADMET compartment where only distribution coefficient (LogD) and partition coefficient (LogP) are slightly above limit while other parameters are with acceptable range.

The SwissADME assessment of Antrocin (structure in Figure 1) as a drug candidate for targeting BRAF/MEK/PI3K complied with the Lipinski rule of five, as well as the Ghose, Veber, Egan, and Muegge standards for drug-likeness, pharmacokinetics (PKs), absorption, distribution, metabolism, and excretion (ADME), and toxicity evaluation of small compounds. Antrocin passed these minimum requirements with a molecular weight of 234.33 g/mol, 0.80 saturation (fraction Csp3), polarity of 26.30 Å^2^ (TPSA), zero rotatable bonds (flexibility), lipophilicity (XLOGP3: 3.44), a solubility score of −3.46 Log S (ESOL), a 55% oral bioavailability score, high GIA, and BBB penetration. The drug-likeness and oral bioavailability profile of Antrocin are said to be optimal because the Csp3 value is greater than 0.25. The low TPSA value depicts that this drug can easily cross the biological barriers like the intestinal barriers since TPSA is less than 140 Å^2^ [65,66] (Figure 11A and Table 2). The pharmacokinetic profiling of Antrocin (Figure 11B) is observed to be within an acceptable range, as it is aligned within the upper and lower limit drug-like parameters. Antrocin is said to be safe and generally drug-like since it passed the required parameters highlighted in Table 2.

### 2.11. Antrocin Is a Potential Drug for Targeting the BRAF/MEK/PI3K Oncogenic Signature

**Figure 12:**
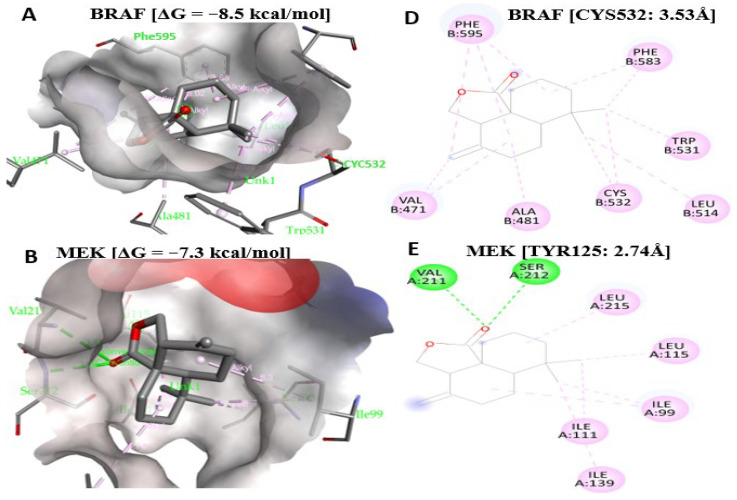

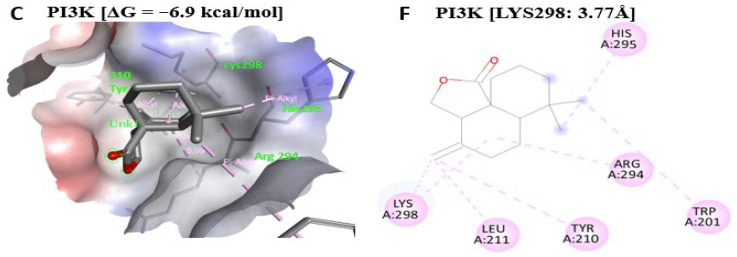
3D and 2D structures of Antrocin’s binding affinity on BRAF, MEK, and PI3K where green indicate hydrogen bonding and purple alkyl bonds. (**A**,**D**) Antrocin binds to BRAF with a lower Gibbs free energy [ΔG = −8.5 kcal/mol]. (**B**,**E**) Binding interaction of Antrocin/MEK [ΔG = −7.3 kcal/mol] on (SER212: 3.00 Å) stabilized by H-bond. (**C**,**F**) Lower binding energy of Antrocin on PI3K [ΔG = −6.9 kcal/mol] stabilized by Alkyl-bond (LYS298: 3.77 Å).

**Figure 13:**
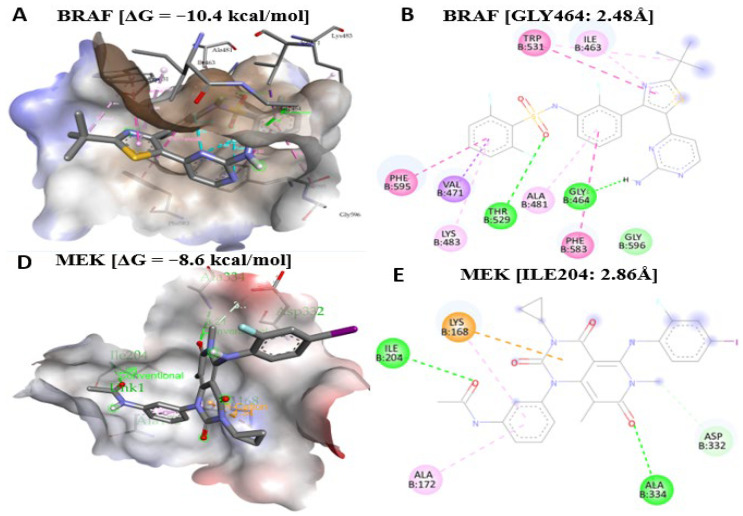

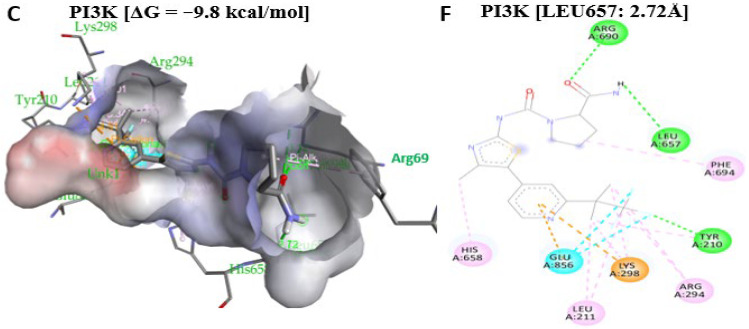
Binding interactions of the standard inhibitors with BRAF/MEK/PI3K, hydrogen bond is indicated green, alkyl bond in purple color, blue for halogen bond while orange shows electrostatic bonding. (**A**,**D**) show a better putative binding of Dabrafenib on BRAF [ΔG = −10.4 kcal/mol] on GLY464 and THR529 stabilized by H-bonds. (**B**,**E**) Trametinib had a slightly better binding energy on MEK [ΔG = −8.6 kcal/mol] on ILE204 (2.86 Å) and ALA334 (3.21 Å) compared to that of the Antrocin/MEK complex. (**C**,**F**) Alpelisib bound to PI3K [ΔG = −9.8 kcal/mol] on LEU657 (2.72 Å), ARG690 (3.23 Å), and TYR210 (3.52 Å).

Ligand-based affinities were utilized in AutoDock Vina to investigate the anti-cancer effects of Antrocin, specifically against the BRAF/MEK/PI3K oncogenic pathway. Antrocin exhibited equally favorable Gibbs free energies in its interactions with BRAF (ΔG = −8.5 kcal/mol), MEK (ΔG = −7.3 kcal/mol), and PI3K (ΔG = −6.9 kcal/mol) (Figure 12A–C), comparable to those of the FDA-approved drugs for these genes. FDA-approved standard inhibitors for BRAF (Dabrafenib), MEK (Trametinib), and PI3K (Alpelisib) were used as a reference for the comparison of Antrocin’s interaction with BRAF/MEK/PI3K [15,61,67,68,69,70,71]. Dabrafenib and BRAF interactions showed ΔG = −10.4 kcal/mol, ΔG = −8.6 kcal/mol for the Trametinib and MEK interaction, and Alpelisib putatively bound to PI3K with ΔG = −9.8 kcal/mol (Figure 13A–C). Furthermore, the interacting amino acids in these interactions and the distance were analyzed and visualized using PyMol and BIOVIA Discovery Studio 2024 Client [72,73,74]. Antrocin and BRAF had no conventional hydrogen bond (H-bond) but with Alkyl (3.53 Å) on CYS532 and Pi-Alkyl bonds. H-bonds for the Antrocin/MEK complex were on SER212 (3.00 Å) and VAL211 (3.12 Å) (Figure 12D,E), while Trametinib had a shorter binding distance to MEK on ILE204 (2.86 Å) and ALA334 (3.21 Å). LYS298 on PI3K formed an alkyl bond with Antrocin at a distance of 3.77 Å, while the Alpelisib/PI3K had a distance of 2.72 Å on LEU657, ARG690 (3.23 Å), and TYR210 (3.52 Å) (Figure 12D–F and Figure 13D–F). This means that Antrocin is a potential anti-cancer drug, as it interacts with BRAF/MEK/PI3K with equally lower Gibbs free energies compared to the standard inhibitors and a short binding distance to the interacting amino acid, indicating its strong binding affinity to this oncogenic signature. These results are illustrated in 3D and 2D structures, along with details of the interacting amino acids, interaction types, binding distances, and energies summarized in Table 3 below.

### 2.12. In Vitro Validation of Antrocin’s CRC Inhibitory Potential Through Inhibition of Multiple Oncogenic Targets

**Figure 14:**
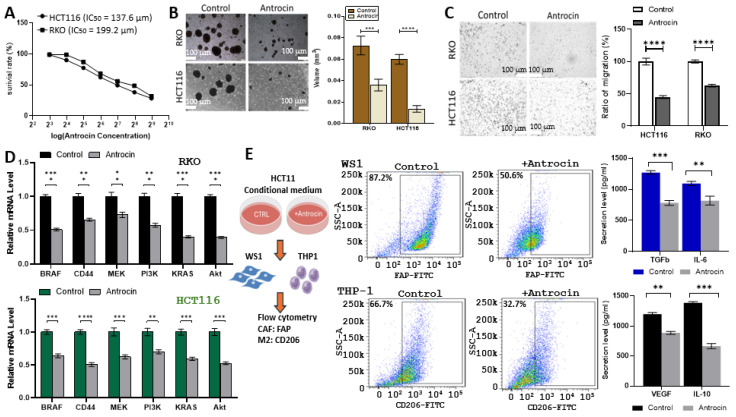
In vitro validation of Antrocin’s anti-CRC functions with higher number of asterisk (*) indicating the higher degree of significance. (**A**) Dose–response curves show cell viability of HCT116 and RKO cells treated with increasing Antrocin concentrations for 48 h; IC50 values are indicated. (**B**) Images and analysis of tumor spheroid formation: cells cultured in ultra-low attachment plates with/without Antrocin; scale bar: 100 μm (**C**) The migration assay shows significant inhibition of HCT116 and RKO cells post-Antrocin; the data represent the migration ratio relative to the control scale bar: 100 μm. (**D**) qPCR shows oncogene expression in HCT116 and RKO cells after Antrocin; mean ± SEM from three experiments; significance levels: * *p* < 0.05, ** *p* < 0.01, *** *p* < 0.001 and **** *p* < 0.001. (**E**) Flow cytometry measures CAF and M2 TAM generation: WS1 fibroblasts and THP-1 cells cultured in conditioned medium from Antrocin-treated or control HCT116; FAP indicates CAF, and CD206 indicates M2 TAM intense blue illustration showing lower ability to generate CAF and TAM; bar graphs show ELISA results for cytokines TGF-β, IL-6, VEGF, and IL-10 quantified.

Based on the bioinformatics analysis, Antrocin was predicted to interfere with the BRAF/MEK/PI3K signaling pathway. Therefore, we performed in vitro studies to validate our hypothesis. Antrocin treatment resulted in a significant, dose-dependent reduction in cell viability in both HCT116 and RKO colorectal cancer cell lines, with IC_50_ values of 137.6 μM and 199.2 μM, respectively (Figure 14A). In three-dimensional tumor spheroid assays, Antrocin markedly impaired spheroid formation, indicating reduced self-renewal and stemness potential in both cell lines (Figure 14B). Additionally, the Transwell migration assay demonstrated that Antrocin treatment significantly reduced the migratory ability of both HCT116 and RKO cells (Figure 14C). Subsequent quantitative PCR analysis showed that Antrocin significantly downregulated the expression of key oncogenic drivers, including BRAF, CD44, MEK, PI3K, KRAS, and AKT, in both HCT116 and RKO cells (Figure 14D), supporting the results of our bioinformatics analysis. To examine whether Antrocin treatment can affect the tumor microenvironment (TME), we cultured WS1 (normal fibroblasts) and THP-1 (macrophage precursors) with the conditioned medium from control and Antrocin-treated CRC cells. Our flow cytometry analysis demonstrated that the conditional medium from Antrocin-treated cells was significantly less capable of generating cancer-associated fibroblasts (CAFs) and M2 tumor-associated macrophages (TAMs), as evidenced by decreased FAP and CD206 expression, respectively (Figure 14E). Supporting this, CAFs and M2 TAMs transformed by the conditional medium from Antrocin-treated HCT116 secreted a markedly reduced amount of TGF-β and IL-6 (CAFs) and VEGF and IL-10 (M2 TAMs), as evident by the ELISA assay (bar graphs, Figure 14E). These findings indicate that Antrocin exerts multifaceted anti-tumor effects by directly inhibiting cancer cell viability, suppressing oncogenic signaling pathways, and disrupting pro-tumorigenic microenvironmental interactions.

### 2.13. Antrocin Treatment Significantly Delays Tumor Growth of HCT116 Tumoroids

**Figure 15:**
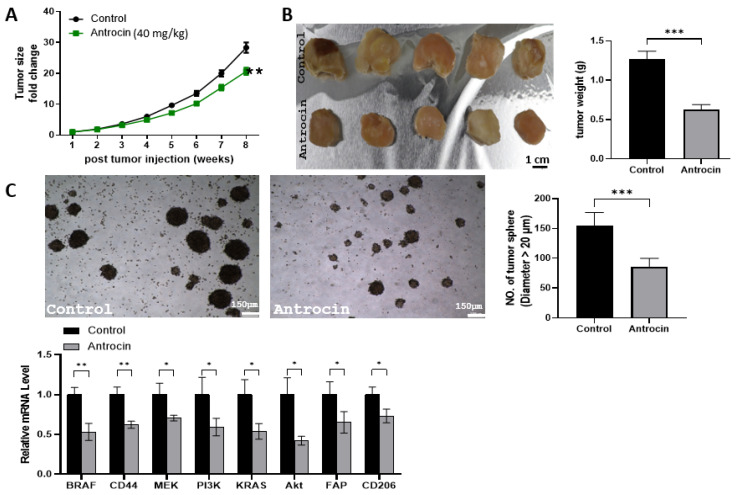
Antrocin shows significant anti-tumor activity in colorectal cancer xenograft models. (**A**) Tumor growth curves demonstrate fold change in tumor size over 8 weeks post-injection in the control (black circles) and Antrocin-treated (green squares, 40 mg/kg) groups. Data represent the mean ± SEM (*n* = 5 animals per group). ** *p* < 0.01. (**B**) Representative photographs of harvested tumors from control and Antrocin treatment groups. Scale bar = 1 cm. Quantitative analysis of tumor weight indicates a significant reduction in the Antrocin-treated animals. Data represent the mean ± SEM. *** *p* < 0.001. (**C**) Tumor sphere formation analysis was conducted using RKO cells isolated from harvested xenograft tumors. Representative images demonstrate reduced sphere formation capacity in the Antrocin-treated group. Scale bar = 150 μm. Quantitative analysis reveals the number of tumor spheres with a diameter greater than 200 μm. Data represent the mean ± SEM from tumor samples. The lower panel presents quantitative PCR analysis of oncogene, CAF, and TAM marker expression in the harvested tumor samples. * *p* < 0.05 and ** *p* < 0.01.

Next, evaluation of Antrocin’s anti-CRC and TME-normalizing functions was performed using the tumoroid-engrafted mouse model. RKO tumoroids (containing CAFs and TAMs) were used for in vivo evaluation of Antrocin. Monitoring tumor growth over 8 weeks demonstrated that Antrocin treatment (40 mg/kg) significantly attenuated tumor progression compared to control groups (Figure 15A). The analysis of tumor size fold change demonstrated a marked divergence between treatment groups starting at week 6 post-injection; tumors treated with Antrocin exhibited delayed tumorigenesis. By week 8, control tumors showed approximately a 28-fold increase in size, while Antrocin-treated tumors indicated only a 20-fold increase, representing a statistically significant reduction in tumor burden (** *p* < 0.01). This pattern of growth inhibition aligns with the compound’s anti-proliferative effects demonstrated in vitro, indicating sustained therapeutic activity throughout the treatment period. Macroscopic examination of harvested tumors demonstrated that Antrocin treatment resulted in notably smaller, less vascularized tumors compared to control animals (Figure 15B). Quantitative analysis of tumor weight revealed a significant reduction in Antrocin-treated animals, with the mean tumor weight decreasing from 1.25 g in the control groups to 0.65 g in the treated animals (** *p* < 0.001). This 48% reduction in tumor weight correlates with the observed growth kinetics and provides additional evidence of Antrocin’s therapeutic efficacy in vivo. Tumor sphere formation assays conducted using cells harvested from the RKO tumoroids showed a marked reduction in sphere-forming capacity in the Antrocin treatment group (Figure 15C). Quantitative analysis indicated that Antrocin-treated tumors generated significantly fewer spheres with a diameter greater than 200 μm compared to control tumors (** *p* < 0.01). This finding suggests that Antrocin treatment effectively suppresses cancer stem cell populations within the tumor mass, potentially reducing the risk of tumor recurrence and metastasis. The expression levels of key oncogenic drivers, including BRAF, CD44, MEK, PI3K, KRAS, AKT, FAP, and CD206, were significantly reduced in Antrocin-treated tumors compared to controls (lower panels, Figure 15C). Notably, the concurrent reduction in FAP and CD206 expression suggests that Antrocin’s effects on tumor microenvironment modulation, initially observed in vitro, are supported in the more complex tumoroid setting. Throughout the 8-week treatment period, Antrocin administration at 40 mg/kg was well-tolerated, with no observable signs of systemic toxicity or significant weight loss in treated animals.

## 3. Discussion

Our initial bioinformatics analysis revealed the overexpression of BRAF, MEK (MAP2K1/2), and PI3K (PIK3CA) in CRC tissues compared to normal tissues. This observation aligns with the growing body of literature highlighting the critical role of these pathways in CRC tumorigenesis. Data mined from The Cancer Genome Atlas (TCGA) indicates that genomic alterations in the RAS/MAPK signaling pathway, including mutations in *KRAS*, *NRAS*, and *BRAF*, as well as in the PI3K/AKT pathway, such as *PIK3CA* mutations, are among the most frequent in colorectal cancer (CRC), collectively occurring in over 50% of cases [48,75,76], and the MAP2K1-K57N alteration all contribute significantly to driving tumor progression and therapy resistance [77,78].

In addition, the mutational landscape of CRC is highly heterogeneous, which is also another major contributor to therapeutic resistance and failure. Our study demonstrates that the interplay between BRAF/MEK/PI3K mutations and the expression of other cancer-related genes provides valuable insight into the complex molecular circuitry of CRC. For instance, the downregulation or mutation in tumor suppressors AXIN2 and APC in BRAF-mutant CRC, as observed in our study, agrees with previous reports linking BRAF mutations to the dysregulation of the Wnt/β-catenin signaling pathway [48,79]. Furthermore, our data revealed that MEK and PI3K mutants lead to the upregulation of genes involved in iron homeostasis (FRRS1) and drug metabolism (SLCO1B3 and CYP4X1), respectively. FRRS1 upregulation, involved in iron metabolism, is increasingly recognized as a critical vulnerability in cancer cells, with elevated iron levels supporting rapid proliferation and DNA synthesis [80,81,82]. The increased expression of drug-metabolizing enzymes, such as SLCO1B3 and CYP4X1, strongly suggests the development of acquired resistance to chemotherapy. These findings indicate that the BRAF/MEK/PI3K oncogenic signature not only drives tumor growth but also contributes to a CRC TME that is more favorable for survival and resistance to therapy.

Equally important, this study demonstrates a significant link between the BRAF/MEK/PI3K oncogenic signature and the establishment of a pro-tumorigenic colorectal cancer microenvironment (TME). The observed correlation between these gene signatures and the infiltration of cancer-associated fibroblasts (CAFs) and M2 tumor-associated macrophages (TAMs) aligns with the established roles of these cell types in promoting tumor growth, angiogenesis, and immunosuppression in CRC [83,84]. Specifically, BRAF V600E-mutant CRC has been linked to a distinct immune microenvironment characterized by increased infiltration of immunosuppressive cells, including M2 TAMs, and a reduced response to immunotherapy [85]. After establishing the oncogenic and TME-shaping roles of the BRAF/MEK/PI3K signaling signature, the in vitro experiments demonstrate that Antrocin can effectively modulate the TME by reducing the differentiation of both CAFs and M2 TAMs. This finding provides further support for Antrocin’s multi-targeted potential, as it suggests that Antrocin not only targets the tumor cells directly but also disrupts the supportive network that they rely on for survival and growth. By creating a less hospitable TME, Antrocin may enhance the efficacy of other therapies, including immunotherapy, and reduce the risk of metastasis. The ability of Antrocin to reprogram the TME represents a key aspect of its multi-faceted anti-cancer activity.

Based on our bioinformatics analysis of the BRAF/MEK/PI3K oncogenic signature and numerous lines of evidence that mono-targeted therapies often fail to improve the outcome of cancer patients, including CRC, due to the development of drug resistance [59]. Thus, identifying compounds with multi-targeting capabilities represents an alternative and emerging strategy for overcoming the limitations of single-agent therapies. We provide in silico support for Antrocin’s drug-likeness and ADME properties, suggesting Antrocin has a favorable pharmacokinetic profile. Molecular docking experiments provide a plausible molecular basis for Antrocin’s multi-targeting activity, demonstrating its ability to form stable complexes with BRAF, MEK, and PI3K, which potentially alter their regular conformation and disrupt their functions. Antrocin belongs to the class of sesquiterpene lactones, which are known to inhibit tumorigenic signaling pathways and induce oxidative stress [86]. Previous studies have demonstrated that Antrocin can inhibit the PI3K/AKT and MAPK signaling pathways in prostate cancer cells, resulting in the induction of apoptosis and suppression of tumor growth [19]. Our findings extend these observations to CRC and provide a more comprehensive understanding of Antrocin’s multi-targeting capabilities. Antrocin’s ability to inhibit both the RAS/MAPK and PI3K/AKT pathways presents a key advantage, as it addresses the two major signaling axes that are dysregulated in CRC.

The translational significance of this study is evident through in vitro and in vivo validation of Antrocin’s effects against CRC. We showed that Antrocin treatment can suppress the tumorigenic properties of HT116 and RKO cell lines, including cell viability, migration, and spheroid formation. Importantly, Antrocin-treated HCT116 and RKO cells exhibited a significantly reduced ability to produce CAFs and M2 TAMs by releasing lower levels of cytokines such as IL-6, TGFb1 (for CAF transformation), and VEGF and IL-10 (for M2 polarization).

Finally, we utilized an RKO tumoroid-engrafted mouse model of CRC (containing CAF and M2 TAM components) to validate Antrocin’s therapeutic potential. The significant delay in tumor growth observed in the Antrocin-treated group, along with no apparent cytotoxicity, provides promising preclinical evidence of its efficacy. Additionally, ex vivo experiments on the tumor cells harvested from the mice demonstrated a significantly reduced spheroid-forming ability in the Antrocin-treated group, accompanied by a decreased expression of the BRAF/MEK/PI3K oncogenic signature, FAP (CAF marker), CD206 (M2 TAM marker), and the stemness marker CD44. Together, the in vitro and in vivo experimental data supported our hypothesis, which was generated by our computational and bioinformatics analysis (as illustrated in Figure 1). The hypothesis obtained from the bioinformatics and experimental analysis of this study gives more clarity for future studies, having observed the mutational sequence, cancer upregulation, cancer hallmark, proliferative signals, relative mRNA levels of these genes in HCT116/RKO cells, and tumor growth of the BRAF/MEK/PI3K signature in colorectal cancer. Also, the multi-target inhibitory potentials of Antrocin in computational, in vitro, and in vivo models create a new avenue to harness further insights on the anticancer potentials of this molecule as well as its mode of action.

## 4. Materials and Methods

### 4.1. BRAF/MEK/PI3K Differential Expression in Colorectal Cancer and Pan-Cancers

Gene differential expressions of the BRAF, MEK, and PI3K signatures in CRC on the Tumor, Normal, and Metastatic plot tool version 1.0 (TNMplot.com) were accessed at https://tnmplot.com/analysis/ on 22 December 2023 [87], using gene chip data for DEG analysis of BRAF, MEK, and PI3K in normal, tumor, and metastatic tissues. The Kruskal–Wallis test compared gene expressions in TNM tissues, followed by Dunn’s test for significant expression of BRAF, MEK, and PI3K with *p* < 0.05. Also, we explored GEPIA2 (http://gepia2.cancer-pku.cn/#index, 22 December 2023) for tissue-wise expression analysis of BRAF, MEK, and PI3K in CRC with *p* < 0.05 and a Log2 fold change cut-off of 2.0 [88]. Information on pan-cancer differential expression of BRAF, MEK, and PI3K was obtained using the PANDA transcriptomic web tool uniroma2 version (https://panda.bio.uniroma2.it/) [89]. Both PANDA and GEPIA2 analyses used cancer data from The Cancer Genome Atlas.

### 4.2. Evaluating BRAF/MEK/PI3K Mutations and Associated Gene Expression Changes in CRC

The relationship between the mutation status of BRAF/MEK/PI3K mutation status and the changes in gene expression in colorectal cancer was analyzed on the muTarget platform (https://www.mutarget.com/, 28 December 2023) at a stringent *p* < 0.01 [90]. The genotype results gave an insight into the genes whose expression will be altered due to the input gene mutation, while the target analysis on this platform unravels the mutation in the genes that will cause changes in the expression of the input gene. Furthermore, the cBioPortal 6.0 version for Cancer Genomics (https://www.cbioportal.org/, 24 April 2024) was used to explore BRAF/MEK/PI3K genomic alterations, particularly driver mutations in CRC and their impact on the patient’s overall survival [91,92,93]. A total of 4334 samples from Colorectal Cancer (MSK, JNCI 2021), Colorectal Adenocarcinoma (TCGA, Pan-Cancer Atlas), Metastatic Colorectal Cancer (MSK, Cancer Cell 2018), Colorectal Adenocarcinoma (DFCI, Cell Reports 2016), and Colorectal Cancer (MSK, Gastroenterology 2020) studies [27,94,95,96] were analyzed.

### 4.3. Network Construction and Pathways Enrichment Analysis in CRC

The Cancer Hallmark and Gene Ontology (GO) pathways of the proposed signaling web were obtained, and further to validate these findings, the human protein atlas was used to ascertain the difference between BRAF/MEK/PI3K in cancerous compared to normal colorectal tissue and ascertain the cell lines inherent. The gene–gene correlation map of the BRAF/MEK/PI3K oncogenic signature was accessed from the Encyclopedia of RNA Interactomes (ENCORI/starBase) Pan-Cancer analysis platform accessed on 4 April 2024 (https://rnasysu.com/encori/index.php) with its gene expression data from the TCGA project [97]. In addition, we analyzed the interactions of these genes and their associated GO biological processes and pathways on the STRING web tool 12.0 (https://string-db.org/) based on the physical interactions, co-expression, predicted co-localization, genetic interactions, pathways, shared protein domains, and molecular signatures database. Furthermore, analysis of the biological processes impacted by the genetic alterations of BRAF/MEK/PI3K in CRC was achieved on the Network Data Exchange (NDEx Cancer Pathways) of the cBioPortal for Cancer Genomics web tool (https://www.cbioportal.org/, 29 April 2024) [91,92]. The analysis focused on only those pathways with a *p*-value < 0.05.

### 4.4. Correlation Between BRAF/MEK/PI3K Expression and Immune Cell Infiltration Levels

This study explored the association of BRAF/MEK/PI3K immune infiltration activities by comparing mutant and wild type using the gene set cancer analysis free version (GSCA) web tool. GSCA comprises a wide range of bioinformatics tools used to comprehensively predict the immune cell infiltration levels in the tumor microenvironment based on the TCGA data (https://guolab.wchscu.cn/GSCA/#/) [98]. This software uses data from 33 cancer types in TCGA, covering over 10 million genomic entries, with ImmuCellAI applied in immunogenomic studies of 24 immune cells. The analysis examined immune infiltration related to BRAF/MEK/PI3K gene sets and single nucleotide variants (SNV groups), using Wilcoxon tests with significance at *p* < 0.01 and FDR < 0.01.

### 4.5. Correlation Between Drug Sensitivity and BRAF/MEK/PI3K Expression in Colorectal Cancer

The Gene Set Cancer Analysis (GSCA) (https://guolab.wchscu.cn/GSCA/#/, 27 December 2023) was also used to determine the correlation between BRAF/MEK/PI3K expression and drug sensitivity in pan-cancer based on both the Genomics of Drug Sensitivity in Cancer (GDSC) and the Cancer Therapeutics Response Portal (CTRP) databases for a comprehensive analysis [98]. The analysis comprised the top 30 FDA-approved drugs, whose half maximal inhibitory concentration (IC_50_) on the expression of BRAF/MEK/PI3K was analyzed using Spearman’s correlation with a statistically significant false discovery rate (FDR) of <0.05.

### 4.6. Chemical Origin and Assessment of the Drug-Likeness, Absorption, Distribution, Metabolism, and Excretion (ADME) Properties of Antrocin

A series of tandem chemical reactions involving 1,7-diynes and internal and external nucleophiles leads to the discovery of drimane-type sesquiterpenoids, an oxygenated stereoselective compound with C-15, one of whose analogues became Antrocin [99]. Pharmacokinetic parameters of Antrocin were explored in the SwissADME version 5 online platform (http://www.swissadme.ch/index.php#top/ 5 April 2024) and ADMETlab version 3.0 (https://admetlab3.scbdd.com/server/screening 26 June 2025) to assess the drug-likeness and medicinal chemistry properties of Antrocin, employing a set of algorithms to evaluate the bioavailability properties, absorption, distribution, metabolism, and excretion (ADME) of Antrocin as a drug candidate [65]. The Pfizer Rule of Five was used to obtain the number of hydrogen donors and acceptors, molecular mass, molar refractivity, and the calculated Log P (CLogP) during our analysis of Antrocin’s drug-likeness [100,101]. Also, the drug’s gastrointestinal absorption (GIA) and ability to cross the blood–brain barrier (BBB) on the Brain and Intestinal Estimated Permeation were analyzed [65].

### 4.7. Molecular Docking Analysis

In silico molecular docking analyses were illustrated using AutoDock Vina commands in the command prompt to predict the binding affinities of Antrocin (CID:53474706) to BRAF mutant (PDB:6V34), MEK (PDB:3SLS), and PI3K (PDB:1E8Y) [72,102,103]. A comparison of the results from these interactions with those of FDA-approved standard inhibitors Dabrafenib (CID:44462760), Trametinib (CID:11707110), and Alpelisib (CID:73265232), which target BRAF, MEK, and PI3K, respectively, was documented. The 3D protein structures of BRAF mutant, MEK, and PI3K in PDB format were downloaded from the RCSB Protein Data Bank (RCSB PDB) (https://www.rcsb.org/, 30 November 2023). All SDF files of the ligands, whose 3D structures were downloaded from PubChem (https://pubchem.ncbi.nlm.nih.gov/, accessed 30 November 2023), were converted to PDB format using Open Babel GUI software version 3.1.1. AutoDock Tools 1.5.7 was used to prepare the ligands and receptors (proteins) for docking, converting them to PDBQT format [72]. Analysis of the docking results was obtained from PyMol and BIOVIA Discovery Studio 2024 Client [72,73,74].

### 4.8. Cell Culture

Human colorectal cancer cell lines HCT116 and RKO, obtained from the American Type Culture Collection (ATCC) (located at 11F.-3, No.350, Minghua 1st Rd., Zuoying Dist., Kaohsiung City 813018, Taiwan), were used for in vitro studies. Cell lines were authenticated using STR analysis (please refer to the Supplementary Materials and Methods Sections Tables S1 and S2). HCT116 is recognized for its high mutational burden, including a KRAS mutation, while RKO is a BRAF-mutated cell line. To examine the impact on the tumor microenvironment (TME), normal human dermal fibroblasts (WS1, Cat #60300, BCRC, Taiwan) and human monocytic leukemia cells (THP-1, Cat #60430, BCRC, Taiwan), which act as macrophage precursors, were utilized. All cell lines were cultured under the conditions suggested by the vendors.

### 4.9. Antrocin Preparation and Treatment

Chemically synthesized Antrocin is a generous gift from Prof. Yew-Min Tzeng (Continuing Education Program of Food Biotechnology Applications, College of Science and Engineering, National Taitung University, Taitung, Taiwan). Antrocin was prepared by dissolving the compound in dimethyl sulfoxide (DMSO) to a concentration of 100 mM as a stock solution. Antrocin was then freshly prepared for each experiment from the stock solution, ensuring that the final DMSO concentration did not exceed 0.1% (*v*/*v*) in any treatment group, including vehicle controls. For cell viability assays, HCT116 and RKO cells were treated with increasing concentrations of Antrocin for 48 h to obtain dose–response curves. For other functional assays, including spheroid formation, transwell migration, and quantitative PCR, a concentration below IC_50_ of Antrocin was used, and vehicle-treated cells received an equivalent volume of DMSO.

### 4.10. Tumor Spheroid Formation Assay

A three-dimensional tumor spheroid formation assay was conducted to evaluate whether Antrocin treatment inhibited the generation of cancer stem-like cells, following our established protocol [104]. In brief, HCT116 and RKO cells were seeded at a density of 10,000 cells/well in ultra-low attachment plates (Corning Pharmaceutical Technologies, Taipei City, Taiwan) in serum-free DMEM/F12 medium supplemented with 20 ng/mL epidermal growth factor (EGF), 10 ng/mL basic fibroblast growth factor (bFGF), and 2% B-27 supplement, with or without Antrocin treatment. Fresh medium was replaced every 2–3 days. Spheroids were allowed to form and grow for 5–7 days. Representative images of the spheroids were captured using an inverted brightfield microscope. Quantitative analysis of spheroid formation was performed by measuring the diameter of at least 10–20 spheroids per well using ImageJ software Fiji version. The scale bar for images was set at 100 μM. Statistical significance was determined by an unpaired *t*-test, with a *p*-value of less than 0.05 considered significant.

### 4.11. Migration Assay

The migration of CRC cells was determined using a Transwell apparatus (Thermo Fisher, Taipei, Taiwan). HCT116 and RKO cells (2 × 10^4^ cells/well) incubated with or without Antrocin (80 μM, 12 h) were seeded into the upper chambers, containing 200 μL serum-free DMEM, and 500 μL DMEM with 10% FBS in the lower chambers, establishing a serum concentration gradient. The cells were cultured for an additional 12 h and were fixed with formaldehyde (10%), followed by crystal violet staining. Cells on the upper side of the membrane were discarded, and cells on the opposite side of the membrane were counted.

### 4.12. Quantitative Real-Time PCR (qPCR) Analysis

Total RNA was extracted and purified using a TRIzol™ Plus RNA Purification Kit (Life Technologies, Taipei, Taiwan). Total RNA (500 ng initially from 5 × 10^5^ cells) was reverse-transcribed using a QIAGEN OneStep RT-PCR Kit (QIAGEN, Taipei, Taiwan), with PCR reactions conducted using a Rotor-Gene SYBR Green PCR kit. The primer sequences utilized in this study are listed in Supplementary Table S3. GAPDH served as a reference for normalization.

### 4.13. Conditioned Medium Preparation and Flow Cytometry

To investigate the effects of Antrocin on the tumor microenvironment (TME), conditioned medium (CM) was prepared from RKO cells that were treated with either Antrocin or vehicle control. The HCT116 cells underwent treatment for 48 h, after which they were washed three times with PBS and cultured in serum-free medium for an additional 24 h. The supernatant was then collected, centrifuged to remove cellular debris, and filtered through a 0.22 µm sterile filter to obtain CM. WS1 cells were incubated with the CM for 72 h to induce differentiation into cancer-associated fibroblasts (CAFs). THP-1 cells were first differentiated into macrophages by treatment with 100 ng/mL phorbol 12-myristate 13-acetate (PMA) for 24 h, followed by incubation with CM for 48 h to induce M2 tumor-associated macrophage (TAM) differentiation.

Cells were harvested using 0.25% trypsin-EDTA, washed twice with PBS, and re-suspended at 1 × 10^6^ cells/mL in staining buffer (PBS containing 2% FBS and 0.1% sodium azide). Moreover, 1 × 10^5^ cells were incubated with primary antibodies at 4 °C for 30 min: FAP monoclonal antibody (clone F11-24, #BMS168, Thermo Fisher Scientific, Taipei, Taiwan) at 1:100 dilutions for cancer-associated fibroblast (CAF) identification and CD206 monoclonal antibody (clone 15-2, Cat. No. MA5-28581, Thermo Fisher Scientific) at 1:100 dilutions for M2 tumor-associated macrophage (TAM) detection. A mouse IgG1 isotype control (Cat. No. PA5-33198, Thermo Fisher Scientific) was used as a negative control. After washing, cells were incubated with an FITC-conjugated secondary antibody (goat anti-mouse IgG, Cat. No. F-2761, Thermo Fisher Scientific) at 1:200 dilutions for 30 min at 4 °C. Flow cytometry was performed using a BD FACSCanto™ II system (BD Biosciences, Taipei, Taiwan), with a minimum of 10,000 events acquired per sample. Data were analyzed using FlowJo™ v11 software. Positive staining was defined as fluorescence intensity exceeding the 99th percentile of the corresponding isotype control.

### 4.14. ELISA Cytokine Profiling

Cytokines secreted by CAFs and M2 TAMs were quantified using enzyme-linked immunosorbent assay (ELISA). Following flow cytometry analysis, differentiated CAFs and M2 TAMs were cultured for an additional 24 h in serum-free medium to allow for cytokine accumulation without interference from serum. Culture supernatants were collected, centrifuged at 2000× *g* for 10 min at 4 °C to remove cellular debris, and stored at −80 °C until analysis. Cytokine concentrations were determined using commercially available ELISA kits according to the manufacturer’s protocols: Human VEGF ELISA Kit (#ab100662), Human IL-10 ELISA Kit (#ab314734), Human TGF-β1 ELISA Kit (#ab100647), and Human IL-6 ELISA Kit (#ab178013) (all from Abcam, Cambridge, UK). All assays followed a double-antibody sandwich format with horseradish peroxidase (HRP) detection. Briefly, 96-well microplates pre-coated with capture antibodies were incubated with standards and samples (100 μL per well) for 2 h at room temperature. After washing, biotinylated detection antibodies were added and incubated for 1 h, followed by incubation with HRP-streptavidin conjugate. Color development was achieved using TMB substrate, and reactions were terminated using sulfuric acid. Optical density (OD) was measured at 450 nm using a CLARIOstar microplate reader (BMG LABTECH, Ortenberg, Germany).

### 4.15. In Vivo Evaluation of Antrocin’s Therapeutic Potential

The mouse experiments performed in this study were approved by the Institutional Animal Care and Use Committee or Panel (IACUC/IACUP) at Taipei Medical University (Approval No. LAC2022-0532). NOD/SCID mice (8-week-old female, BioLASCO, Taipei, Taiwan) were injected with RKO tumoroids (generated from the 3D co-culture of RKO, WS1, and THP-1 cells in a ratio of 10:3:1) [105,106]. Approximately 5 × 10^6^ cells/tumoroids suspended in a 1:1 mixture of serum-free medium and Matrigel (Corning, Taiwan) were subcutaneously injected into the right flank of each mouse. When the tumors reached an average volume of approximately 100 mm^3^ (2 weeks post-injection), the mice were randomized into two groups (*n* = 5 animals per group): a control group receiving a sham injection and a treatment group receiving Antrocin (40 mg/kg, intraperitoneal injection, 5 times a week). Tumor growth was monitored every week using standard caliper measurements. Tumor volume was calculated using the formula: Volume = (L × W^2^)/2. The tumor size fold change was calculated relative to the initial tumor volume at the start of treatment. After the 8-week treatment period, mice were humanely euthanized by cervical dislocation. Tumors were harvested and weighed. Harvested tumor samples were then processed for further analysis.

### 4.16. Statistical Analyses

All statistical analyses were performed using GraphPad Prism software (version 8.42). Data are presented as the mean ± standard error of the mean (SEM) unless otherwise specified. For comparisons between two groups, an unpaired Student’s *t*-test was employed. For dose–response curves and IC_50_ calculations, non-linear regression analysis was used.

## 5. Conclusions

Using a combined bioinformatics and experimental approach, we quickly identified the BRAF/MEK/PI3K oncogenic signature as a key therapeutic target for CRC. Antrocin’s ability to modulate this signature and the TME was validated using preclinical models, and our results laid the foundation for Antrocin’s future clinical translation. Although HCT116 and RKO cell lines were utilized in this study, further research can showcase the effect of Antrocin on several other CRC cell lines, as well as other CRC signaling axes aside from BRAF/MEK/PI3K. That notwithstanding, this study is a significant step towards harnessing other anticancer inhibitory potentials of Antrocin in colorectal cancer.

## Figures and Tables

**Table 1:** Tabulated cancer hallmarks of BRAF/MEK/PI3K showing significant *p*-values.

Term	*p*-Value	Adj. *p*-Value	Genes
Sustaining proliferative signaling	0.013518	0.015019988	BRAF/MAP2K1/PIK3CA
Genome instability	0.1420924	0.14209238	MAP2K1
Evading growth suppressors	0.0105248	0.013155949	BRAF/MAP2K1/PIK3CA
Evading immune destruction	0.000124	0.000372266	BRAF/MAP2K1/PIK3CA
Sustained angiogenesis	0.0001489	0.000372266	BRAF/MAP2K1/PIK3CA
Tissue invasion and metastasis	0.0036863	0.005551356	BRAF/MAP2K1/PIK3CA
Tumor-promoting inflammation	0.0001342	0.000372266	BRAF/MAP2K1/PIK3CA
Resisting cell death	0.0021638	0.00432761	BRAF/MAP2K1/PIK3CA
Reprogramming energy metabolism	0.0001196	0.000372266	BRAF/MAP2K1/PIK3CA
Replicative immortality	0.0038859	0.005551356	MAP2K1/PIK3CA

**Table 2:** A summary of physicochemical properties, pharmacokinetics, drug-likeness, and medical chemistry of Antrocin.

Formula and SMILE of Antrocin	Physicochemical Properties	Aqueous Solubility	Pharmacokinetics and Absorption	Drug-Likeness	Toxicity
Formula: C_15_H_22_O_2_ SMILE: CC1(C)CCCC23C(COC2=O)C(=C)CCC13	Molecular weight: 234.33g/mol NHA: 2 NHD: 0 NRB: 0 Molar Refractivity: 68.17 Lipophilicity: 3.31	Log S (Ali): −3.67 Log S (ESOL): −3.46 Log S (SILICOS-IT): −3.52 Class: Soluble	BBB permanent: Yes GI absorption: High TPSA: 26.30 Å^2^ (high crossing of biological barriers) Bioavailability score:0.55 CYP2C19: No CYP1A2: No CYP2C9:Yes	Lipinski: Yes Egan: Yes Veber: Yes Muegge: Yes Ghose: Yes	hERG blocking: (0–0.1): poor Rat acute toxicity (0.1–0.3): poor Genotoxicity: poor

**Table 3:** Detailed molecular docking results of Antrocin on BRAF/MEK/PI3K. The tabulated docking results consist of the Gibbs free energies of this interaction, the interacting amino acid on the receptor, binding distances, and bond types involved.

Proteins and Gibbs Free Energy	Protein Interaction Site and Bonding Proximity	Bond Type
BRAF [ΔG = −8.5 kcal/mol]	CYS532 (3.53 Å), ALA481, VAL471, LEU514	Alkyl
	TRP531, PHE595, PHE583	Pi–Alkyl
MEK [ΔG = −7.3 kcal/mol]	SER212 (3.00 Å), VAL211 (3.12 Å)	Conventional hydrogen
	LEU115, LEU215, ILE99, ILE111, ILE139	Alkyl
PI3K [ΔG = −6.9 kcal/mol]	LYS298 (3.77 Å), LEU211, ARG294	Alkyl
	HIS295, TRP201, TYR210	Pi–Alkyl

## Data Availability

All the generated and analyzed datasets of this study are available upon reasonable request.

## Supplementary Materials

The following supporting information can be downloaded at: https://www.mdpi.com/article/10.3390/ijms26188780/s1.

## Abbreviations

CRC	Colorectal cancer
TME	Tumor microenvironment
BRAF	B-Raf proto-oncogene, serine/threonine kinase
MEK	Mitogen-activated protein kinase kinase (MAPK/ERK kinase)
PI3K	Phosphoinositide 3-kinase
MAP2K1	Mitogen-activated protein kinase kinase 1 (MEK1)
PIK3CA	Phosphatidylinositol-4,5-bisphosphate 3-kinase catalytic subunit alpha
GSCA	Gene Set Cancer Analysis (integrated cancer genomics platform)
