# Supplementary material for: Integrated In Silico and Experimental Validation of Antrocin as a Plant-Derived Multi-Target Therapeutic for BRAF/MEK/PI3K-Driven Colorectal Cancer
Source: Int J Mol Sci. 2025 Sep 9;26(18):8780. doi: 10.3390/ijms26188780 (PMC12469948; doi:10.3390/ijms26188780)
Supplement: Supplementary file 1 [file ijms-26-08780-s001.zip › ijms-3793415-supplementary.pdf]

Integrated *In Silico* and Experimental Validation of Antrocin as a Plant-Derived Multi-Target Therapeutic for BRAF/MEK/PI3K-Driven Colorectal Cancer

Supplementary data

Human Cell Line Genotyping Report (Table S1 and S2)

Cell lines were authenticated using STR analysis by Topgen Biotechnology Co., Ltd

Address: 11F.-3, No.350, Minghua 1st Rd., Zuoying Dist., Kaohsiung City 813018, Taiwan, Taiwan • +886 7 5565212 tel +886 7 5565122 fax. topgen.com.tw

Materials and Methods:

DNA Extraction by general total DNA isolation Kit (DNA conc.: 1374 ng/uL, OD260/280: 2.1) for RKO cells.

DNA Extraction by general total DNA isolation Kit (DNA conc.: 588 ng/uL OD260/280: 2.1) for HCT116 cells.

The multiplex PCR is performed by using the AmpFLSTR Identifier PLUS PCR Amplification Kit (Applied Biosystems, USA) containing 16 STR loci. The PCR products labeled with different fluorescence are analyzed with GeneMapper ID v3.1 on the capillary Genetic DNA analyzer 3730 (Applied Biosystems, USA). The genotyping results of the sample are searched in the human STR profile database. The DSMZ, together with the ATCC, JCRB, RIKEN, ECACC, and ExPASy repositories, including data sets of more than 2,455 cell lines, has generated comprehensive databases of short tandem repeats (STR) cell line profiles.

Table S1: RKO cell line authenticity analysis showing 97.7% similarity score.

Results ：Matched to RKO Cell line.

	Locus names								
	D5S818	D13S317	D7S820	D16S539	VWA	TH01	AM	TPOX	CSF1PO
	11,13	8,11	8,10,11	11,12,13	16,17,22	6,10	X,X	10,11	8,10

Database:

ATCC
DSMZ
JCRB
RIKEN
ECACC
ExPASy

Similarity Score	Cell line	D5S818	D13S317	D7S820	D16S539	vWA	TH01	AM	TPOX	CSF1 PO
	Your query	11,13	8,11	8,10,11	11,12,13	16,17,22	6,10	X,X	10,11	8,10
97.7%	RKO	11,13	8,11	8,10,11	11,12,13	16,17,22	6,10	X,X	9,10,11	8,10

Searching the genotypes of the sample in the ATCC / DSMZ / JCRB / RIKEN/ ECACC / ExPASy STR database, the test sample result matched to RKO Cell line.

Table S2: HCT 116 cell line authenticity analysis showing 88.9% similarity score with Luc2 11.1%. These HCT116 cells will be used for our ongoing investigation of Antrocin’s anti-metastasis potential; hence, Luc2 (luciferase gene) can be used for non-invasive molecular imaging to track metastatic HCT116-Luc cells.

Results ：Matched to HCT 116-Luc2 Cell line.

	Locus names								
	D5S818	D13S317	D7S820	D16S539	VWA	TH01	AM	TPOX	CSF1PO
	10,11	10,12	11,12	11,13	17,22	8,9	X,X	8,8	7,10

Database:

ATCC
DSMZ
JCRB
RIKEN
ECACC
ExPASy

Similarity Score	Cell line	D5S818	D13S317	D7S820	D16S539	vWA	TH01	AM	TPOX	CSF1PO
	Your query	10,11	10,12	11,12	11,13	17,22	8,9	X,X	8,8	7,10
100%	HCT 116-Luc2	10,11	10,12	11,12	11,13	17,22	8,9	X,X	8,8	7,10
88.9%	HCT 116	10,11	10,12	11,12	11,13	17,22	8,9	X,Y	8,9	7,10

Searching the genotypes of the sample in the ATCC / DSMZ / JCRB / RIKEN/ ECACC / ExPASy STR database, the test sample result matched to HCT 116-Luc2 Cell line.

Table S3: qPCR primer list

Gene	Forward	Reverse
BRAF	AACTCTTCATAATGCTTGCTCTGA	CAGACAACTGTTCAAACTGATGGGACC
CD44	CCCAGATGGAGAAAGCTCTG	ACTTGGCTTTCTGTCCTCCA
MEK	GCTTCTATGGTGCGTTCT	GAGTTGACTAGGATGTTGGA
PI3K	AGTAGGCAACCGTGAAGAAAAG	GAGGTGAATTGAGGTCCCTAAGA
KRAS	TGGACGAATATGATCCAACAAT	TCCCTCATTGCACTGTACTCC
Akt	TCACCTCTGAGACCGACACC	ACTGGCTGAGTAGGAGAACTGG
FAP	GGAAGTGCCTGTTCCAGCAATG	TGTCTGCCAGTCTTCCCTGAAG
CD206	TTGCACTTTGAGGGAAGGGA	CCTTGCCTGATGCCAGGTTA
GAPDH	CATCATCCCTGCCTCTACTG	GCCTGCTTCACCACCTTC
